# Obstetric care providers are able to assess psychosocial risks, identify and refer high-risk pregnant women: validation of a short assessment tool – the KINDEX Greek version

**DOI:** 10.1186/s12884-015-0462-y

**Published:** 2015-02-21

**Authors:** Andria Spyridou, Maggie Schauer, Martina Ruf-Leuschner

**Affiliations:** University of Konstanz, Constance, Germany; Vivo international (www.vivo.org), Constance, Germany; Department of Psychology, University of Konstanz, Clinical Psychology & Behavioral Neuroscience Unit, Post Box 905, Constance, D-78457 Germany

## Abstract

**Background:**

Prenatal assessment for psychosocial risk factors and prevention and intervention is scarce and, in most cases, nonexistent in obstetrical care. In this study we aimed to evaluate if the KINDEX, a short instrument developed in Germany, is a useful tool in the hands of non-trained medical staff, in order to identify and refer women in psychosocial risk to the adequate mental health and social services. We also examined the criterion-related concurrent validity of the tool through a validation interview carried out by an expert clinical psychologist. Our final objective was to achieve the cultural adaptation of the KINDEX Greek Version and to offer a valid tool for the psychosocial risk assessment to the obstetric care providers.

**Methods:**

Two obstetricians and five midwives carried out 93 KINDEX interviews (duration 20 minutes) with pregnant women to assess psychosocial risk factors present during pregnancy. Afterwards they referred women who they identified having two or more psychosocial risk factors to the mental health attention unit of the hospital. During the validation procedure an expert clinical psychologist carried out diagnostic interviews with a randomized subsample of 50 pregnant women based on established diagnostic instruments for stress and psychopathology, like the PSS-14, ESI, PDS, HSCL-25.

**Results:**

Significant correlations between the results obtained through the assessment using the KINDEX and the risk areas of stress, psychopathology and trauma load assessed in the validation interview demonstrate the criterion-related concurrent validity of the KINDEX. The referral accuracy of the medical staff is confirmed through comparisons between pregnant women who have and have not been referred to the mental health attention unit.

**Conclusions:**

Prenatal screenings for psychosocial risks like the KINDEX are feasible in public health settings in Greece. In addition, validity was confirmed in high correlations between the KINDEX results and the results of the validation interviews. The KINDEX Greek version can be considered a valid tool, which can be used by non-trained medical staff providing obstetrical care to identify high-risk women and refer them to adequate mental health and social services. These kind of assessments are indispensable for the promotion of a healthy family environment and child development.

## Background

Research advances in the area of prenatal psychology have shown that many psychosocial factors in addition to medical risks have a severe impact not only on the general well-being of the pregnant woman but also on the fetus. Brain development starts to take place in the first months following conception, while the first three years of life are very important on a neurological level [1,2]. Psychological and social risk factors existing during the prenatal period increase the risk of adverse obstetrical, neonatal and postnatal outcomes. Their persistence into the postnatal period compromises positive mother-child interaction and presents further challenges to the child and their emotional, behavioral and social development [2,3] as well as neuropsychological development [4,5]. Nevertheless, the transfer of this research knowledge into practice only began in the past decade [6,7]. Worldwide, there are only a few studies reporting the development, evaluation and implementation of screening tools for psychosocial risk factors in pregnant women and subsequent intervention and prevention programs in community health centers in the U.S. [8], Australia [9] and Canada [10].

*Psychopathology*, especially depression, is one of the best-known psychosocial risk factors during pregnancy. It has been associated with a wide variety of medical problems in the mother, as well as with other psychosocial risk factors such as stress and domestic violence [11]. Depressed pregnant women are more likely to have preterm birth [12] and are at higher risk for suffering adverse obstetric outcomes [13,14] and low birth weight [4]. Additionally, depression in pregnant women is the strongest predictor of poor psychological wellbeing [15] and for lower maternal-fetal attachment (MFA) [16]. Not only depression, but general maternal psychopathology during the prenatal period is associated with shorter gestational length and lower infant size on birth [17]. Infant developmental outcomes are negatively affected by prenatal exposure to antidepressants, and maternal depressed mood in pregnancy [18].

Beside psychopathology, *prenatal stress* is another crucial risk factor for pregnant women. Latest studies have shown that prenatal maternal stress is related to alterations in the maternal plasma cortisol and amniotic fluid cortisol; these changes may eventually induce negative birth outcomes and detrimental infant emotional development [19]. Nevertheless, the adverse effects of early exposure to high levels of glucorticoid on infant cognitive development may be moderated by the quality of maternal-infant attachment [20]. Long term effects of stress experienced in pregnancy has also been shown to increase risk of behavioral problems linked to altered the Hypothalamic Pituitary Adrenal Axis (HPA) activity [21]. Associations between prenatal stress and offspring birth weight, gestational age and antisocial behaviour were found in mother–offspring pairs to be consistent [22].

Women from *low socioeconomic status* [23]*, adolescent or very young mothers (<21 years of age)* [24], *immigrant* [25] are more likely to present higher level of perceived stress while *refugees* from war-torn societies are often diagnosed with PTSD [26,27]. These social groups often lack social support [28], a stress mediating factor [29], present higher levels of IPV, drug abuse [30] child maltreatment and present worse parenting skills [31]; all the above conditions result in poorer birth outcomes [32-34].

*MFA* is a crucial predictor for later maternal-child attachment. Positive MFA was found to be a protective factor against depressive symptoms both during pregnancy as well as up to 6 months after birth [35]. Positive associations are found also with good health practices such as abstinence from smoking, alcohol and drug abuse [36]. Therefore negative is considered as a serious risk factor with strong associations to further psychosocial risks.

*Intimate partner violence (IPV*) obviously is another risk factor that if present prior and during pregnancy causes pregnancy complications, adverse perinatal and neonatal outcomes [37] and breastfeeding problems [38,39]. Experiencing domestic violence during pregnancy has been associated with depression, anxiety and PTSD symptoms in the perinatal period [40]. Additionally, children whose mothers were exposed to IPV during pregnancy are more likely to suffer physical violence during childhood [41]. Studies even have revealed that the methylation status of the GR gene in adolescent children is influenced by maternal experience of IPV during pregnancy [42].

*Maternal Childhood Abuse (MCA) and lifetime PTSD* in the mother are also related to postpartum depression, impaired maternal-child attachment, worse mental health during pregnancy [43] and poorer mother-child interactions [44]. These type of traumatic experiences are frequently unidentified risk factors for the appearance of depressive and post-traumatic stress symptoms in pregnant women [45]. MCA is also associated with children’s poor behavioral trajectories and more negative events in childhood such as physical abuse, separation from parents and changes in family composition [46]. Mediated pathways have been found between MCA to maternal substance abuse and offspring victimization, perpetrating the vicious cycle of violence [47,48].

Similarly, *maternal childhood sexual abuse (MCSA)* is related to postpartum anxiety and depression in adolescent mothers [49]. Several studies indicate that women that suffered sexual abuse in childhood are also more likely to be re-victimized in adulthood by their partners and at the same time suffer more PTSD symptoms [50,51]. But, the most crucial impact of such experiences are the long-term repercussions for adult mental health, parenting, and child adjustment in the succeeding generation [52]. Women that suffered adverse childhood experiences (ACEs) are also more prompt to become adolescent mothers, while negative psychosocial status and fetal deaths regularly attributed to adolescent pregnancy seem to result from underlying ACEs rather than adolescent pregnancy per se [53].

Even though the review on prenatal existing psychosocial risk factors and their detrimental effects on mother and child given here cannot be fully comprehensive, it becomes obvious that early identification of these factors during the sensitive prenatal period is the key in preventing adverse outcomes for mother and child. But so far only a few projects worldwide are found where prenatal assessment was introduced into the practice of midwives and gynecologists. In the current literature we observe an overall, comprehensive lack of prenatal tools that address multiple psychosocial risk factors [54].

Johnson et al. [55] in a review of the existing tools for factors influencing perinatal mental health assessment revealed 6 valid instruments. This review assessed the reliability, validity, sensibility and specificity and normative data when these were reported by the authors. The results revealed that tools where assessing factors from 3 domains [Contextual Assessment of Maternity Experience (CAME)], to 26 [Camberwell Assessment of Need—Mothers (CAN-M)]. All the assessment tools were *‘not recommended*’ due to the existence of ‘unacceptable’ reliability, validity or normative data based on the Hammil scoring system.

In Australia, prenatal screening measures were integrated in the midwifery practice and were generally accepted well by both midwives and pregnant women; as a result high-risk women were identified and referred to social workers. The psychosocial risk assessment model (PRAM) using the Edinburgh Depression Scale and the psychosocial risk-based Antenatal Risk Questionnaire (ANRQ) embedded in the integrated perinatal care context at the Royal Hospital for Women in Sydney, Australia on 2,142 women, were used to compute a Psychosocial Risk Index (PRI) in order to guide individualized care planning [6]. Recently, a shorter version of the ANRQ was applied after extracting 12 items from the original 23 item-Pregnancy Risk Questionnaire to assess how the shorter ANRQ would perform. Johnson et al. [55] found this tool to fulfill more of the requirements than any of the others assessed. It assesses seven psychosocial risk domains: emotional support from subject’s own mother in childhood, past history of depressed mood or mental illness and treatment received, perceived level of support available after birth of the baby, partner emotional support, life stresses in the past 12 months, personality (anxious or perfectionist traits) and history of abuse (emotional, physical and sexual). It has a rating score from a minimum of 5 to a possible maximum of 62 and the authors suggest a clinically relevant cutoff of 23. The psychometric properties of the tool include acceptable sensitivity (0.62) and specificity (0.64), it has high face and construct validity of the factors assessed, and has high acceptability amongst midwives and pregnant women, nevertheless it has low positive and negative predictive values [55]. It is a key component in early identification of mental health risks and morbidity across the perinatal period [9].

Despite some published research on the development and evaluation of prenatal psychosocial risk factors, the literature is not without its limitations. The need for longitudinal research examining the predictive validity of the tools for child development is outstanding. The majority of the tools embedded in the prenatal care used by health professionals is reported from western English-speaking cultures. In the literature we could not find reports of similar screening protocols in countries such as Greece, that due to the impact of the financial crisis in the EU zone is struggling to maintain its social stability.

To corroborate this we had an exploratory field assessment through interviews with the medical staff working in the public health services with pregnant women in the island of Crete. Through this we could verify the gap in the prenatal care related to psychosocial risks’ screening.

Relevant information on the Greek health system in the Health in Transition (HiT) report published by the European Observatory on health Systems and Policies describes the Hellenic Health System (ESY) as ‘*a mixture of public integrated, public contract and public reimbursement models, comprising elements from both the public and private sectors and incorporating principles of different organizational patterns.*’ Health care is provided from both private and public centers, although the first is more frequently observed in primary care and pharmaceuticals [56]. Greek population is aging constantly due to decrease of fertility rates in the past thirty years; birth rates are stretching to equality with death rates, counting 10.5 births per 1000 population while life expectancy is of 80.1 years on average and 82.1 for women. Mortality among women is 46 deaths per 1000 female adults (15–60 years) and infant mortality 2.7 per 1000 live births [56].

Greek women are in the first place among 40 countries for daily tobacco smoking reaching 34%, [57]. Latest data related with illicit drug use in Greece from 2004 show a 8.6% prevalence among the population of lifetime use, while another study in 2006 in the cities of Athens, Heraklion and Thessaloniki showed that 14% of the females had, at some point, tried an illicit drug [58].

As far as financial means are concerned, poverty rates in Greece were higher than the average EU rates even before the economy entered a severe crisis in 2009. Latest Eurostat reports on unemployment bring Greece first in the Eurozone with rates reaching 27.2% and 59.1% among young people (<25 years) [59]. The deep economic recession that befell Greece in 2009 triggered the interest of the scientific community on the impact of the financial crisis on mental health; yielding to studies showing a link between major depression [60] and suicidality escalation [61] to the economic hardship. Related data to perinatal mental health state are scarce, but a study carried out in Athens in 2008 showed that 19.8% of the participants suffered postpartum depression during the first 6 months after delivery, while this was found to be related to stressful events during pregnancy, among other factors [62].

In terms of cultural context Greece is a collectivist culture [63,64] where family is the core of emotional and material support and where people rely on their families’ support. In the case of Crete, “development” and “modernization” is attributed to the development of the tourism industry. Despite the acute changes at a societal, civil and cultural level observed in the past decades, Cretans still consider their communities as small states where the extended family unit is still the central point of reference and commitment [64].

Considering the encouraging results of projects using prenatal assessment tools for psychosocial risks in other countries and their importance in the prevention of adverse effects on mother-child relationship and child development, the KINDEX interview was developed by Schauer and Ruf-Leuschner after a critical review of evidence-based literature [65]. The KINDEX is a brief instrument developed originally in German, designed to be used by medical staff such as gynecologists, obstetricians, midwives and counselors in prenatal-perinatal health care. Bearing in mind the current situation in Greece and the financial pressures that may impact women’s health and increase risk factors in pregnant women together with the lack of any other prenatal assessment of psychosocial risks, we were driven to adapt the KINDEX in the Greek language and proceed to implement it in primary attention settings in Crete.

### Aim of this study

Our study has four aims. First, we wanted to explore whether the use of the KINDEX is feasible in the daily practice of medical staff providing prenatal care in Greece in a representative sample of the general population.

Second, we wanted to evaluate if the KINDEX is a useful tool in the hands of non-trained medical staff, in order to identify and refer high-risk women to the adequate mental health and social services.

Third we wanted to examine the criterion-related concurrent validity of the KINDEX by assessing the relation of the KINDEX interview with the validation interview carried out by an expert clinical psychologist.

The final objective was to achieve the cultural adaptation of the KINDEX Greek Version and to offer a valid tool for the psychosocial risk assessment to the obstetric care providers.

## Methods

### Translation procedure – KINDEX

The translation procedure was based on the World Health Organization guidelines for translation process and adaptation of instruments [66]. This was achieved through the following steps: 1) forward translation by two bilingual health professionals familiar with both the German and Greek cultures. 2) An experts’ panel formed by bilingual health experts and translation/adaptation experts who agreed on the adequacy of the translated version. 3) Back translation was done by two independent bilingual translators with emphasis on the conceptual and cultural equivalence. Discrepancies found were delimited and agreement by the expert’s panel was achieved after small changes. 4) We discussed with the medical staff about the adequacy of the items and when all items were completely clarified between the translators and the medical staff the translation procedure was completed. The same procedure was followed for those instruments included in the validation interview that had not been used in the Greek population.

### Setting: time and place of the interviews

All interviews were carried out in the region of Chania, Crete, in four different health centres. 1) Sixty interviews (64.5%) were carried out in the external consultation of the Gynecological Department of the Saint George Hospital of Chania. 2) Nineteen (20.4%) were carried out in the Internal Unit of the Saint George Hospital of Chania where women with pregnancy complications were hospitalized. 3) Ten (10.8%) were carried out in the Medical-Social Center of (PIKPA) Chania while attending maternal preparation classes and 4) four interviews (4.3%) in the Health Center of Vamos village, a primary attention center for the population of eleven villages of the Chania region. Fourteen (15.1%) women were interviewed exclusively by midwives in the Medical-Social Center of Chania while attending maternal preparation (MP) classes. No significant difference was found in the KINDEX sum score between women who were interviewed in the external consultation (*M* = 4.83, *sd* = 2.62, *range* = 1-13), those who were hospitalized (*M* = 4.52, *sd* = 2.77, *range* = 0-10), those who were interviewed in the Medical-Social Centre (*M* = 5.10 *sd* = 3.14, *range* = 1-13), and the ones interviewed in the Vamos Health Centre (*M* = 4.0 *sd* = .86, *range* = 3-10), [*H*(3) = .72; *p* = .86]. All KINDEX interviews were carried out between November 2011 and March 2012.

The study received ethical clearance from the Ethics Committee of the University of Konstanz, Germany which decided in accordance with the principles of the Declaration of Helsinki (in the version of Seoul 2008).

### Interviewers

KINDEX: Seventy women (75.3%) were interviewed by five midwives and twenty-three (24.7%) by two gynecologists. No significant difference in the KINDEX sum score was found between women that were interviewed by gynecologists (*M* = 4.62 *sd* = 2.45, *range* = 0-13), and those interviewed by midwives (*M* = 5.17 *sd* = 3.25, *range* = 1-13), (*U* = 772.5; *p* = .77). Interviewers participating in the study did not receive any financial or other kind of compensation and participated in the study voluntarily.

Validation Interview: All interviews were carried out by a psychology PhD-Student using standardized assessment instruments. The student was trained in the use of all instruments previously in the Competence Centre of Psychotraumatology in the Psychology Department of the University of Konstanz and fluent in Greek. Participating medical staff did not receive any financial compensation for the time invested in conducting the interviews.

Drop-outs were not reported by the interviewers during the study, while the medical staff reported that nine participants refused to participate in the KINDEX interview without giving any justification of their decision while this was not requested by the medical staff.

### Procedure

Interviewers were instructed to either ask all pregnant women coming for their appointment, MP class or hospitalization to follow a fixed, randomized procedure^a^ when, due to time constraints, it was not possible to ask all of them to participate. In most of the cases the medical staff invited all the pregnant women who fulfilled the requirements to participate in the study. Inclusion in the study required the participants to have good comprehension skills of the Greek language and have a gestational age between 10–33 weeks. We chose this period considering that women very early in their pregnancy (first 1–3 months) and at the end of it (last 1–2 months) would have more limitations attending their appointment or MP class due to possible hyperemesis in the first case [67,68], or driving restrictions in the second. Interviewers had to use the KINDEX to interview the participants and were instructed not to administer it as a self-report questionnaire. The participant was required to read the information sheet and give her written consent in order to proceed with the interview. The participants interviewed with the KINDEX did not receive any compensation but the subsample that participated in the Validation Interview did receive 15€ for their participation. All medical staff collaborating in the project attended a short information session, organized by the psychology Phd-student coordinating the study, regarding the eleven risk factors assessed by the KINDEX. An instructional information sheet along with a colorful “KINDEX” template, indicating the items referring to each risk factor, was introduced and distributed and the medical staff was encouraged to use when making referral decisions to the Mental Health Department in the hospital. The department was made up of one psychiatrist, two psychologists and two social workers. The PhD student coordinating the study had a meeting with the Mental Health Department as well, in order to give the necessary information about the study and the possibility that some pregnant women would be referred to the Department.

Women were informed that if after the KINDEX assessment felt the need to talk with a psychologist they would have the opportunity to do so, at the Mental Health department of the Hospital. During the assessment they were again informed about this possibility through a text that was read to them prior the items concerning experiences of child abuse, sexual of physical, were addressed. Referral criteria in a similar study, using an assessment of 12 possible risk factors, was the presence of one factor, nevertheless in the assessment they did not include factors such as the immigrant status of the parents or possible financial difficulties [7]. Since these factors are included in the KINDEX we considered that it would be an overestimation to consider a woman at risk solely due to her immigrant status or that of her partner, especially in a city where a high number of immigrants are integrated in the society. We decided to set the referral criteria at two risks and more, since this could increase the possibilities of identifying true cases and differentiating women of low and high risk [69]. Midwives were also advised to make these referrals in conjunction with their own clinical judgment and if they considered that the presence of just one serious risk factor (e.g. illegal drug consumption, or possibility for current unattended psychopathology) was present they were encouraged to refer the participant. The KINDEX software application for tablets and smartphones (developed later than this study) gives an immediate feedback of the results to the interviewer and suggests possible measures to be taken, such as referring the pregnant woman [70]. In future studies the use of this software will enable an accurate identification of those women at risk based on the KINDEX.

All interviews took place in a private room where no other family members were allowed. During the KINDEX interviewing procedure a psychology PhD-student of the Clinical Psychology department of the University of Konstanz was reachable and had weekly meetings with the medical staff to discuss interviewing progress and to collect the completed KINDEX questionnaires.

Ethical clearance for the study was provided by the Ethics Committee of the University of Konstanz according to the Helsinki Declaration^b^.

### The instrument: KINDEX

The KINDEX was developed at the University of Konstanz, Germany in 2009 [71] based on the current literature on risk factors for healthy child development. Thirty-four items that assess 11 different risk factors compose the KINDEX.

The first risk factor found in the KINDEX is *mother’s age*, which uses an ordinal scale. Using the age range a binary item was created. Mother’s age of 21 and younger is considered to be a risk factor (see Table 1, risk area 1). The decision on the age was taken after considering that studies indicate that in maternal age younger than 21 higher rates of postpartum hemorrhage and higher rates of low 5-min Apgar scores could occur [72] while the risk that adolescent pregnancy entail for birth, and child outcomes are described above.

**Table 1:** **Overview of the risk areas, scales, number of items and the risk definition**

	**Risk area**	**Number of items**	**Scale**	**Definition as a risk**	**Items included in the KINDEX sum score**
1	Age	1	Ordinal	≤21	1
2	Migration	2	Binary	Immigration mother or father	2
3	Single parent	1	Binary	Single parent	0^1^
4	Financial problems	2	Binary	Worry about financial problems	2
			Binary	Housing index ≤ 0.5 (rooms/person)	
5	Physical symptoms, complications, medical risks	3	Binary	Physical Symptoms, complications, medical risks	3
6	Complicated prenatal bonding	5	Binary	Unplanned Pregnancy	5
			Ordinal	Concerns 7–10 (mother and father) Joy 0–3 (mother and father)	
7	Current stress	4	Ordinal	PSS-4 sum score ≥ 12	1
8	Traumatic experiences during childhood	2	Binary	Physical abuse	2
				Sexual abuse	
9	Intimate partner violence (IPV)	4	Binary	Increasing number of disputes; vociferous fights in the past 8 weeks; fights including physical violence in the last 8 weeks; physical violence in a past relationship.	4
10	Substance abuse	6	Binary	Nicotine, alcohol, drugs/mother and father.	5^2^
11	Mental illness	4	Binary	Ever-psychiatric diagnosis, inpatient treatment, psychotropic drugs, asked for help (psychotherapy or counseling center).	3^3^

Note: ^1^The item is excluded from the reliability analysis, all the women lived with their partners, ^2^the item for mothers’ drug use is excluded from the reliability analysis, none of the participants was consuming illicit drugs, ^3^the item for inpatient treatment is excluded none of the participants was ever inpatient in a psychiatric clinic.

*Migration* is another risk factor that was measured through two binary items (mothers and fathers history of migration) (see Table 1, risk area 2).

The factor of the *“single parent”* for the mother is also recoded dichotomously (see Table 1, risk area 3).

The two items, *financial difficulties* and *housing situation* compose the financial problems factor. The financial difficulties item is binary and the housing situation is recoded by the number of rooms per person. Consequently a housing situation index of 0.5 or less is regarded to be a risk factor (see Table 1, risk area 4).

*Physical symptoms*, complications during pregnancy and *medical risk factors* are assessed through three binary questions (see Table 1, risk area 5).

*Prenatal attachment* is assessed through five items. A binary item regarding the planning of the pregnancy measures prenatal attachment. An unplanned pregnancy is assumed to be a negative factor. In addition, the mother and father’s joy and worries about the future with their baby is recorded on a 0 to 10 scale. The items of joy and worries are recoded into a binary scale, the upper (worries) and lower (joy) third are considered to be negative prenatal attachment. Therefore “joy” in the range of 0–3 as well as “worries” in the range of 7–10 are considered to be factors of negative prenatal attachment (see Table 1, risk area 6).

*Perceived current stress* as experienced by the pregnant woman is measured through an ordinal scale, the PSS-4 (Perceived Stress Scale) [73]. The PSS-4 is a standardized instrument that collects, through a four-item Likert-scale, (0–4) the current perceived stress level. It is the abbreviated version of the PSS-14 used in the validation interview, which uses 14 items. A sum score is calculated for the scale, where the maximum total value is 16. We transformed the scale into a dichotomized variable. Thus, the upper quartile is assumed to be a load factor of high-perceived stress (total score ≥ 12) (see Table 1, risk area 7).

*Traumatic experiences during childhood* are levied through two binary questions concerning physical or sexual abuse during childhood and adolescence (see Table 1, risk area 8).

*Violent and stressful experiences within intimate partner relationship* are assessed through four binary questions (i.e. increase in fighting, vociferous fights in the last 8 weeks, fights with physical violence past 8 weeks, previous relationship with IPV) (see Table 1, risk area 9).

*Substance abuse* (smoking, alcohol, drugs) is also recorded for the pregnant woman and the father through three binary questions each. When a question is positively answered, there is the option to specify the kind and quantity of substance, though this information is not included in the analysis (see Table 1, risk area 10).

*Mental health* is assessed through four binary questions (lifetime history of psychiatric diagnosis, inpatient therapy, psychotropic drugs treatment, asked for psychological help). The option to specify is also given here, but again it is not included in the analysis. The questionnaire concludes with an open question concerning mother’s wishes for support during pregnancy and for the future with the baby (see Table 1, risk area 11).

Calculating Cronbach’s alpha was achieved after recoding the ordinal scales into binary as described above. Some of the component variables had zero variance and were excluded from the calculation of Cronbach’s alpha. Three variables were excluded from the reliability analysis because they had zero variation: “single parent”, (all women lived with their partner), “conflicts involving physical violence in the past eight weeks” (none of the women reported physical violence) and “previous psychiatric hospitalization” (none of the participants ever received psychiatric inpatient treatment). Therefore, the analysis consisted of 27 variables: maternal age, migration of the mother, migration of the father, worries about financial difficulties and housing index, physical violence during childhood, sexual abuse during childhood, relationship problems and interpersonal violence (3 items) mother's substance use (smoking/alcohol), substance use by the father (smoking, alcohol, drugs), mental illness history (3 items), medical risk factors (3 items), prenatal bonding (5 items), perceived current stress (PSS4). The Cronbach’s alpha was *α = .67* for 27 items in the KINDEX.

### Validation interview

The validation interview consists of different standardized and half-standardized tools. All the instruments were applied in a clinical interview and by an experienced psychologist and not as self-reports.

Sociodemographic information was collected through half-standardized questions created to assess age, working situation of parents, marital state, previous and current pregnancy as well as the self-reported health condition of the participant.

Stress was assessed through the Perceived Stress Scale (PSS-14; Cohen, Kamarck, & Mermelstein, [73]) which measures the subjective perception of stress. The items are related to the last month and are rated on a 5-point Likert scale ranging from 0 = never to 4 = very often. PSS-14 scores are obtained by re-coding the scores of seven positive items and afterwards summing all 14 items. Possible scores range from 0–56. The Greek Version of the PSS-14 was validated in two studies in the general Greek population [74,75]. Strong internal consistency (Cronbach’s *α* = 0.85) as well as moderate-to-good concordance between psychodiagnostic interview of stress and PSS − 14 (Kendall’s tau-b = 0.43, *p* < 0.01) was observed. Reliability analysis for our sample revealed a Cronbach’s alpha of .81.

In addition to the PSS-14, the Everyday Stressors Index (ESI) [76] was used. A validated version in Greek was not found, therefore we followed the procedure of back-translation with bilingual translators to obtain the Greek Version that was then used in this study. The ESI consists of 20 items on a 4-point Likert Scale ranging from 0 (not bothered at all) to 3 (bothered a great deal). It assesses the areas of financial concerns, congestion, job problems, child rearing and interpersonal conflicts. A composite score derives by summing responses to all items. Possible scores range from 0–60. Reliability analysis for our sample revealed a Cronbach’s alpha of .82.

The “global stress” value was created by summing the z-transformed sum scores of the stress scales (PSS14 & ESI).

To assess experiences of family violence during childhood, we used the Checklist of Family Violence, an instrument used in previous studies in different countries and cultures [77,78]. A validated version in Greek was again not found therefore we followed the procedure of back-translation with bilingual translators to obtain the Greek Version that was used in this study. The questionnaire consists of five subscales that assess *physical abuse, verbal-emotional abuse, sexual abuse, witnessed violence* and *neglect during childhood*. The scores for each scale are obtained by summing across items and then all the scales’ scores were summed up to calculate the overall sumscore of the CFV. Reliability analysis for our sample revealed a Cronbach’s alpha of .78.

*Traumatic events and post-traumatic stress symptoms* were assessed by the Posttraumatic Stress Diagnostic Scale (PDS) [79]; the scale is intended to screen for the presence of PTSD in patients who have identified themselves as victims of a traumatic event or to assess symptom severity and functioning in patients already identified as suffering from PTSD. It has four sections. Part 1 is a trauma checklist. Part 2 is the PTSD diagnostic interview, which based on the A1 and A2 criterion according to DSM-IV. Part 3 assesses the 17 PTSD symptoms. It consists of 17 items rating the severity of the symptom from 0 (“not at all or only one time”) to 3 (“5 or more times a week/almost always”). Part 4 assesses interference of the symptoms with all day functioning. The PDS yields a total symptom severity score (ranging from 0 to 51) that largely reflects the frequency of the 17 symptoms of PTSD [80]. The Greek version of the instrument that was already applied in a study with the Greek population, was used in this study [81]. Reliability analysis for our sample revealed a Cronbach’s alpha of .84.

The “global trauma-load” value was calculated by summing the z-transformed sum score of traumatic experiences (PDS-events) and the z-transformed sum score of experiences of family violence (CFV).

Various other instruments were used in addition to assess *psychopathology symptoms.* For the assessment of anxiety and depression, the Greek version of the Hopkins Symptom Checklist 25 (HSCL-25) [81,82] was used. The Greek Version was translated and culturally adapted for studies carried out previously in Greece [83]. It consists of 25 items: Part I of the HSCL-25 consists of 10 items assessing anxiety symptoms; Part II consists of 15 items assessing depression symptoms. Each item can be rated on a Likert Scale ranging from 1 (“Not at all,”) to 4 (“Extremely”). Two scores are calculated: the anxiety score is the sum score of the 10 items and ranges from 10 to 40, while the depression score is the sum score of the 15 depression items and ranges from 15 to 60. In general, the validity of the instrument is well established and there is evidence supporting good test-retest reliability for anxiety (*r* = .75) and depression (*r* = .81). Both scales demonstrated high internal consistency in our sample (Cronbach’s α = .84 for anxiety and α = .86 for depression).

To assess *somatization* symptoms we used the somatization subscale of the Spanish Version of the SCL-90-R [84] which consists of 12 items rated on a 5-point Likert-Scale, ranging from 0 = not at all, to 4 = extremely. The Greek version of the SCL-90 used was validated in a study with psychiatric patients reporting sensitivity of 0.98 and specificity of 0.74 in indicating active psychiatric patients [85] and used later on in a study of the Greek population affected by both the wildfires and earthquake in 2007 and 2008 respectively in the Peloponnese’s area [86]. The score is calculated by summing across the 12 items and possible scores can range from 0–48. The somatization scale of the SCL-90-R demonstrated high internal consistency (Cronbach’s α = .84) for our study’s sample.

To calculate the “global psychopathology” value, we summed the z-transformed sum score of the somatization subscale (SCL-90), z-transformed sum score of the depression and anxiety scales (HSCL-25) and the z-transformed sum score of posttraumatic symptoms (PDS-symptoms).

In Table 2 we present all the means (m), ranges (min-max), standard deviations (sd) of all the measures described above.

**Table 2:** **Means, (±SD) of the sample in the variables assessed in the validation interview**

**Scale**	**N**	**M**	**SD**	**Mdn**	**Min**	**Max**
PSS-14 (Stress)	50	25.88	4.71	19.0	1	36.0
ESI (Stress)	50	29.14	7.13	26.0	20	57.0
Global stress	50	.00	1.74	-.35	−3.30	6.11
HSCL-depression	50	6.20	5.62	5.0	0	24.0
HSCL-anxiety	50	4.04	4.68	3.0	0	19.0
SCL-somatization	50	10.62	8.56	8.0	0	37.0
PDS-PTSD symptoms	50	2.13	3.74	.00	0	18.0
Global psychopathology	50	-.06	3.15	-.96	−3.42	9.49
CFV (Child maltreatment)	50	2.83	3.46	1.00	0	15.0
PDS (Traumatic events)	50	1.92	1.52	2.00	0	5.0
Global trauma load	50	.001	1.70	-.48	−2.08	4.87

Note: N (number of participants), M (mean), SD (standard deviation), Mdn (Median), Min (score minimum), Max (score maximum), PSS-14 (perceived stress scale-14 items), ESI (everyday stress index), HSCL (hopkins symptoms checklist) SCL, (symptom checklist), PDS (posttraumatic stress diagnostic scale), CFV (checklist of family violence).

### Sample

Participants were pregnant women that came to the external consultation of their regular doctor’s appointment in the Gynecological Department of the Saint George Hospital and the Health Centre of Vamos; women that were hospitalized due to pregnancy complications and women that were attending maternal preparation classes in the Medical-Social Center of Chania. Ninety-three pregnant women with an average age of 31 years (range: 20–44, *SD* = 5.34) and gestational weeks average of 18 (range: 10–33, *SD* = 5.34) participated in the study. Fourteen (15.9%) participants were not born in Greece. Detailed sample description as collected from the KINDEX is presented in Table 3.

**Table 3:** **Overview of the risk factors in the KINDEX**

**Load factors**	**Item**	**N**		**KINDEX**	**KINDEX and validation**	**Only KINDEX**	**p**
	Gestational age	93	M (SD)	17.72 (5.95)	17.62 (5.98)	17.84 (5.98)	ns
Alter	Age in years	93	M (SD)	30.7 (3.80)	30.80 (5.17)	30.58 (5.59)	ns
Migration	Mother	93	N (%)	14 (15.1%)	5 (10%)	9 (20.9%)	ns
	Father	93	N (%)	15 (16.1%)	7 (14%)	8 (18.6%)	ns
Single parent	Not living with the father	93	N (%)	0	0	0	ns
Financial worries	Housing index ≤ 0,5 (Room/Person)	93	N (%)	10 (10.8%)	3 (6%)	7 (16.3%)	ns
	Financial worries	93	N (%)	53 (57%)	30 (60%)	23 (53.5%)	ns
Physical complaints and medical risk factors	Physical complaints	93	N (%)	39 (41.9%)	22 (44%)	17 (39.5%)	ns
	Complications	93	N (%)	15 (16.1%)	10 (20%)	5 (11.6%)	ns
	Medical risk factors	93	N (%)	7 (7.5%)	2 (4%)	5 (11.6%)	ns
Prenatal bonding	Unplanned Pregnancy	93	N (%)	35 (37.6%)	18 (36%)	17 (39.5%)	ns
	Joy Mother (0 to 10)	93	M (SD)	9.03 (1.67)	8.78 (1.85)	9.33 (1.41)	ns
	Worries mother (0 to 10)	93	M (SD)	5.59 (3.23)	5.70 (2.77)	5.47 (3.73)	ns
	Joy father (0 to 10)	93	M (SD)	9.29 (1.64)	9.20 (1.91)	9.40 (1.27)	ns
	Worries father (0 to 10)	93	M (SD)	5.28 (3.39)	5.18 (3.28)	5.40 (3.56)	ns
Stress	PSS-4 sum score	93	M (SD)	4.50 (3.15)	4.36 (3.28)	4.67 (3.02)	ns
Abuse in childhood	Physical maltreatment	93	N (%)	15 (16.1%)	4 (8%)	11 (25.6%)	.02
	Sexual abuse	93	N (%)	5 (5.4%)	2 (4%)	3 (7%)	ns
Intimate partner conflict and violence	Increase in conflicts (past 8 weeks)	93	N (%)	12 (12.9%)	7 (14%)	5 (11.6%)	ns
	Vociferous fights (past 8 weeks)	93	N (%)	10 (10.8%)	7 (14%)	3 (7%)	ns
	Fights involving physical violence (past 8 weeks)	93	N (%)	0	0	0	ns
	Ever violent intimate partner relationship	93	N (%)	2 (2.2%)	2 (4%)	0	ns
Nicotine, alcohol and drugs	Smoking (pregnant)	93	N (%)	14 (15.1%)	8 (16%)	6 (14%)	ns
	Alcohol (pregnant)	93	N (%)	8 (8.6%)	5 (10%)	3 (7%)	ns
	Drug consumption (mother)	93	N (%)	0	0	0	ns
	Smoking (father)	93	N (%)	42 (45.2%)	22 (44%)	20 (46.5%)	ns
	Alcohol (father)	93	N (%)	16 (16.1%)	9 (18%)	7 (16.3%)	ns
	Drug consumption (father)	93	N (%)	1 (1.2%)	1 (1.2%)	0	ns
Psychiatric history	Ever psychiatric diagnosis	93	N (%)	9 (9.7%)	8 (16%)	1 (2.3%)	.02
	Ever psychotropic medicine	93	N (%)	3 (3.2%)	3 (6%)	0	ns
	Ever inpatient psychiatric treatment	93	N (%)	0	0	0	ns
	Ever asked for psychological help	93	N (%)	27 (29%)	18 (36%)	9 (20.9%)	ns
KINDEX	KINDEX Sum Score	93	M (SD)	4.76 (2.66)	4.24 (2.82)	4.13 (2.67)	ns

Sample description and differences in risk reports between the group who participated in the KINDEX and in the validation interview and the group that only participated in the KINDEX.

Note: Crosstabs was used for the items scored on dichotomous scales. Parametric *t*-test was used for the ordinal scales KINDEX and Validation: Group that participated in the validation interview, Only KINDEX: Group that only participated in the KINDEX Interview.

### Statistical analysis

Statistical analysis was performed using SPSS 21^st^ Version. Sum scores of the instruments’ scales used in the validation interview were *z*-transformed and *z* values were summed up to create three global values. The “global trauma-load” value was calculated by summing the z-transformed sum score of traumatic experiences (PDS-events) and the z-transformed sum score of experiences of family violence (CFV).

We explored the normality assumption through the Kolmogorov-Smirnov normality test, for the global stress, global psychopathology and global trauma load values. The K-S test values were *D*(50) = .418; *p* = .99, for the global stress, *D*(50) = .704; *p* = .70 for the global psychopathology, *D*(50) = .1.39; *p* = .06 for the global trauma-load and *D*(50) = .20; *p* ≤ .001 for the KINDEX Sum score. The significant value (≤.005) that resulted from the K-S test indicates that the normality assumption is not met for the KINDEX while the other three scales were normally distributed. Therefore we calculated Spearman correlation coefficient to explore correlates between the variables.

To examine risks’ frequency reported by our sample in the KINDEX interview, we performed descriptive statistics.

Kruskal Wallis H test was conducted in order to assess whether there were differences in the KINDEX sum score depending on the public health unit where the interviews were carried out.

In order to examine if there were differences between the randomly selected sub-group that participated in both the KINDEX interview and the validation interview and the group that only participated in the KINDEX interview, we used Chi Square to compare the frequency of the risks in the two groups (e.g. number of immigrants in each group). To compare the means for each group in the linear scales of the KINDEX and the KINDEX sum score, we used non-parametric Mann Whitney *U* test.

We examine the referral accuracy of the medical staff through comparisons between the participants that were referred and those that were not with regard to the KINDEX (Mann–Whitney *U* test) sum score and the global scores of stress, psychopathology and trauma-load (independent samples *t*-test).

## Results

Between the sample that participated only in the KINDEX and the subsample that participated in both the KINDEX and the validation interview only two significant difference was observed: Eight of nine participants who ever received a psychiatric diagnosis were involved in the validation interviews while only one person with a history of a psychiatric diagnosis was not involved (see Table 3). Similarly, eleven of the women that reported child physical abuse participated only in the KINDEX interview while four participated in both the KINDEX and the Validation Interview (see Table 3).

### Concurrent validity: correlations between the KINDEX sum score and the global scores in the validation interview

**Figure 1:**
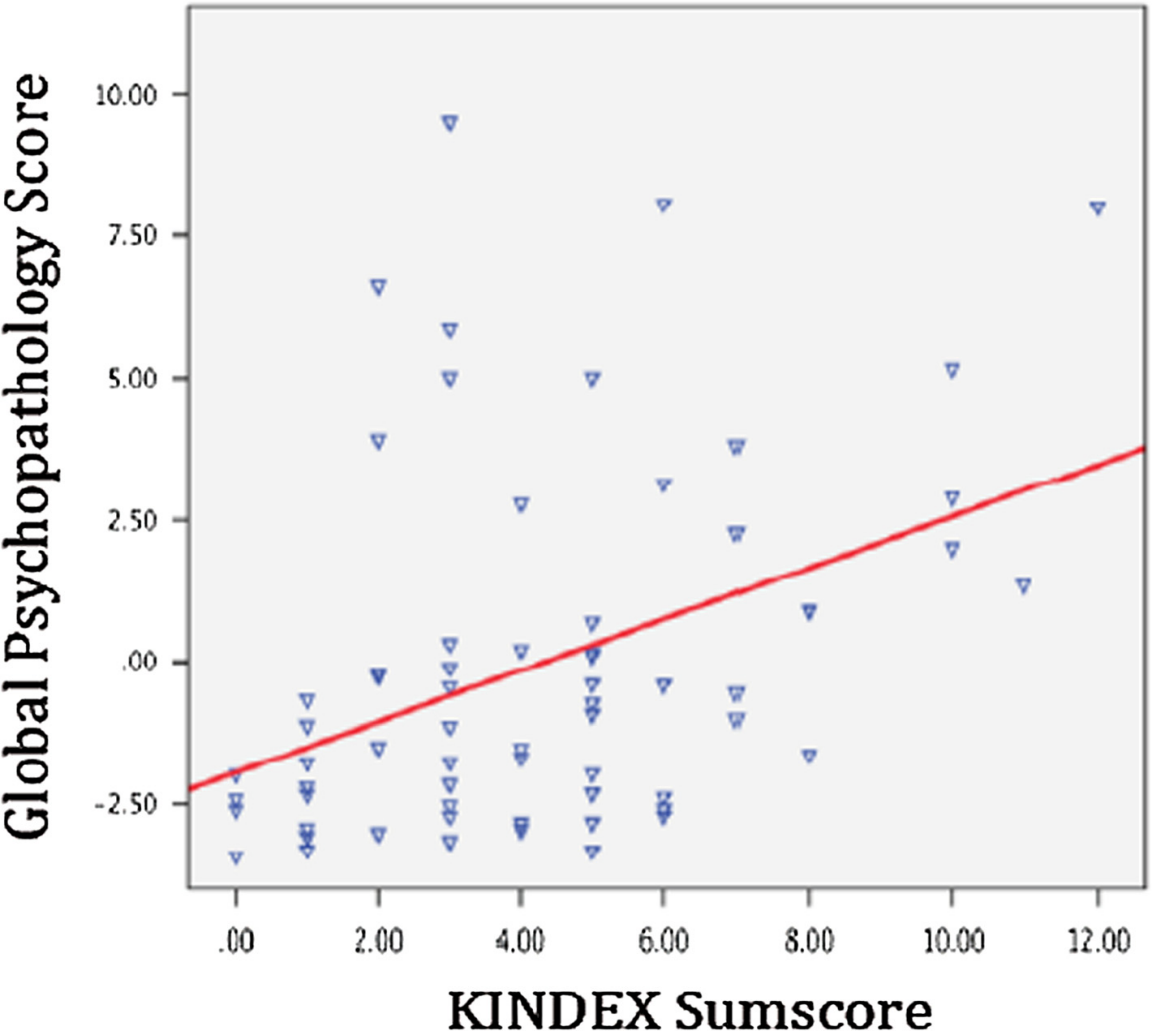
**Relation between the KINDEX sum score on the X-axis and the global psychopathology score (left Y-axis).**

**Figure 2:**
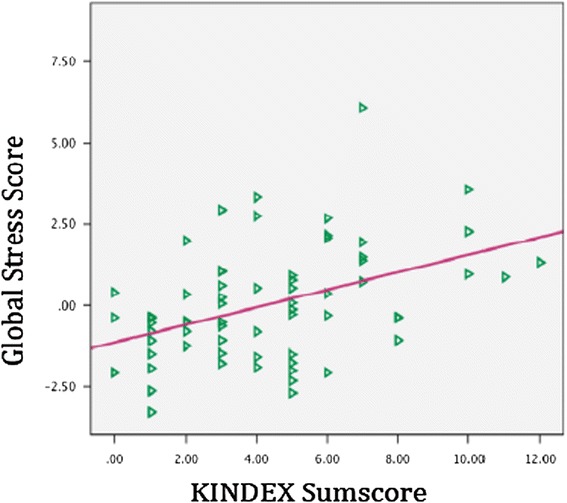
**Relation between the KINDEX sum score on the X-axis and the global stress score (left Y-axis).**

**Figure 3:**
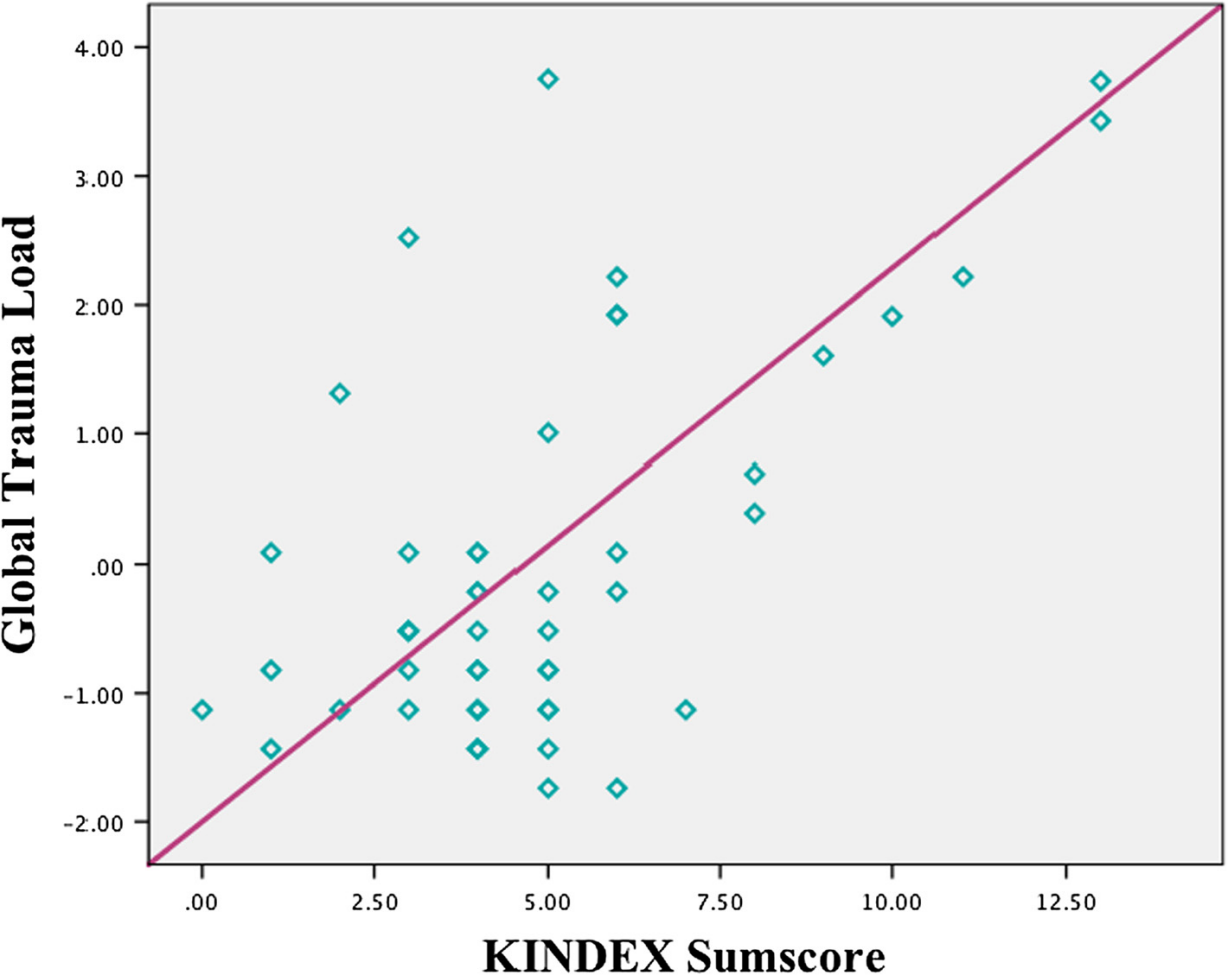
**Relation between the KINDEX sum score on the X-axis and the global trauma load (left Y-axis).**

To examine the concurrent validity of the KINDEX, a sum score was calculated including the 27 dichotomous items (*Mdn* = 4.0, *M* = 4.76, min = 0, max = 13, and *SD* = 2.66) (see Table 1). The sum score was then correlated with the global stress value, the global trauma-load value and the global psychopathology value as assessed in the validation interview. The KINDEX sum score correlated positively with the global stress score (*r* = .44; *p* ≤ .001), the global psychopathology score (*r* = .61; *p* ≤ .001) the global trauma load score (*r* = .59; *p* ≤ .001)) (see Table 4 & Figures 1, 2, 3).

**Table 4:** **Correlates between the KINDEX and the global stress, global psychopathology, and the global trauma load in the validation interview**

	**KINDEX sum score**	**Validation global stress score**	**Validation global psychopathology score**	**Validation global trauma load**
	**N = 93**	**N = 50**	**N = 50**	**N = 50**
KINDEX sum score	1	.45**	.44**	.38**
Validation		1	.62**	.27*
Global stress score				
Validation			1	.45**
Global psychopathology score				
Validation				1
Global trauma load				

Note: *Significance is important in the level of ≤.0.05. **Correlation significant in the level of ≤ .001.

### Referral accuracy: in-group comparisons between referred and non-referred women with regard to the KINDEX sum score and global stress, global psychopathology and global trauma load

Thirteen of the study participants were referred by the medical staff to the mental health attention unit of the hospital. Nine of them also participated in the validation interview. As illustrated in Figure 2, non-parametric Mann–Whitney Test revealed that participants who were referred had higher average KINDEX sum scores (*Mdn* = 9, min = 5, max = 13) than participants that were not referred (*M* = 4.0, min = 0, max = 9), (*U* = 50.5; *p* ≤ .001).

Non-parametric independent samples Mann–Whitney test showed that participants that were referred also show significantly higher medians in the global stress score (referred: *Mdn* = 1.36, range = 7.09, not referred: *Mdn* = −.29; range = 5.78 *U* = 93.0, *p* ≤ .02), in the psychopathology score (referred: *Mdn* = 3.16, range = 12.95; not referred: *Mdn* = −.73; range = 8.07; *U* = −66.0, *p* ≤ .003) and the trauma load (referred: *Mdn* = 1.84, range = 4.25; not referred: *Mdn* = −.40; range = 5.49; *U* = 39.50, *p* ≤ .001) in the validation interview (see Figure 2.)

## Discussion

The present study had four main aims a) to investigate the feasibility of the KINDEX Greek version used by non-trained medical staff providing obstetrical care in public health settings in Greece, b) to assess medical staff’s referral accuracy of women burdened by different risk factors to Mental and Social services based on the KINDEX interview and c) to test the concurrent validity of the instrument.

Our results indicate that the KINDEX has high feasibility used by non-trained medical staff in public health settings in Greece. This is supported through the low drop rates of both the participants and the medical staff during the study and through the positive feedback received through the experience of the medical staff and the participants. The referral accuracy is demonstrated high as shown by the higher scores in the global values of psychopathology, stress and trauma for those women that were referred by the medical staff. Significant positive correlations between the KINDEX sum score and the global values assessed in the validation interview indicate high concurrent validity of the KINDEX.

Recent studies reported efforts to incorporate monitoring in the prenatal period for psychosocial risks. Generally, the results indicate that targeted assessments using instruments to identify women of higher psychosocial risk within primary care settings increases early detection and places women in need in the pipeline for more adequate assistance and resources [6,8,9,87,88].

In our study untrained medical staff using the KINDEX to conduct interviews with pregnant women in their daily clinical practice reported experiencing no problems in carrying out the interviews throughout the study. In order to achieve an optimal feasibility assessment in the public health sector, we chose to not introduce extreme modifications to the daily routine of the collaborating medical staff. None of the midwives or gynecologists dropped-out from the study before completion, even though they did not receive any financial or other compensation for the extra work they conducted. In general, the involvement of their patients in the interview was also very successful, since no dropouts were registered once the women were began the study. The medical staff reported a total of nine women that refused to participate in this study when they were invited do so. No justification for their refusal to participate was required. In addition, during the validation interviews pregnant women frequently reported that even though the KINDEX interview was an unexpected health service, that afterwards they felt more cared for by the medical staff and stated that these kind of questions made their medical treatment more humanized and patient-centered. Therefore, we consider that the feasibility of the KINDEX Greek version in a public health setting established.

Following the instructions the medical staff received, they referred thirteen women to mental health services. With great satisfaction, we could confirm that their referral –even not based on a sum score resulting from the KINDEX – was accurate based on the scores later calculated: a) the KINDEX sum score and b) the three global scores in the validation interview*.* Our results revealed that women who were referred (*n* = 13) had higher KINDEX sum scores and higher global scores in all three risk-areas (psychopathology, stress and trauma load) in the validation interview, than women that were not referred (*n* = 80). Based on the fact that the medical staff was able to make referral decisions immediately after the interview was completed and without having to compute a sum-score, provides some evidence that interviewers using the KINDEX gain insight into the psychosocial state of their patients. In terms of referral accuracy, we conclude that non-trained medical staff, with minimal instruction, is able to identify and refer pregnant women in need to adequate support services after the KINDEX interview in an informal meeting.

In current literature we find many studies reporting specific assessment of psychological risks, especially depression [89], anxiety and distress [90]. Some studies have included a broader range of psychosocial and medical risks in assessments applied in the perinatal period [6,7,9,91] while fewer have suggested interventions with high-risk women [87,90]. The majority of these studies have been developed in the USA, Australia and Canada, while in Europe there are no identified studies applying perinatal assessments for psychosocial risks. In the assessment reported by Matthey et al. [7], 12 items assessing psychosocial risks and the Edinburgh Depression Scale (EDS: measuring depression symptoms severity) were applied covering seven risk areas; this assessment revealed that 12% of the women had three or more of these risk domains. Similarly, in our study we found that 14% were referred to mental health services by the medical staff using the KINDEX. The difference between this assessment and the KINDEX interview is that the KINDEX covers a boarder range of risks (11 risk factors) and that it does not assess symptom’s severity since all risk factor are weighted equally. This seems to be the case for several studies using the EDS as a complement to the assessment tools that give greater importance to perinatal depression over other risks [6,7,91]. In this study we aimed to address the overall pregnant population without excluding groups due to nationality, age or socioeconomic status.

In Greece no previous studies assessing psychosocial risks in the prenatal period using a standardized structured interview instrument were found. Therefore, in order to test the concurrent validity of the KINDEX we were unable to apply an established instrument measuring the exact same concepts. As a result, our validation interview conducted by a clinical psychologist included standardized instruments for three major risk areas linked to many other psychosocial risk factors namely *stress*, *trauma load* and *psychopathology.* For validation, we took a randomized subsample of women initially interviewed with the KINDEX. Correlations between the KINDEX and the global stress, global psychopathology, and global trauma load were moderately high and positive (see Table 4) establishing the concurrent criterion-related validity of the KINDEX. Based on this, we conclude that the KINDEX can be used to assess eleven psychosocial risks related to the three main risk areas (psychopathology, stress, trauma-load) in public health settings. Similar results confirming the criterion-related concurrent validity of the KINDEX were found in previous validation studies following the same methodology in Germany [71], Spain [92] and Peru [93].

## Conclusions

Overall, we conclude that the KINDEX is feasible for use in the Greek public health sector, that referrals made by medical staff based on the KINDEX interview are accurate and the validity of the KINDEX Greek version is given. Clinical implications of these results are significant, since transferring this knowledge in the development of new health policies would definitely improve obstetrical care and ameliorate and prevent long-run serious complications in child development and health. As a result, maternal-child relation and global family wellbeing can be fostered. In addition to prenatal assessments like the KINDEX, low-threshold but evidence-based intervention programs have to be developed and specifically tailored to the special needs of pregnant women burdened by different risk factors. In combination, the KINDEX and low-threshold programs for women in need could be a cost-effective and present revolutionary progress for preventive medicine. This combination could have a significant impact on primary care, creating a more integrative comprehensive health attention towards pregnant women, neonates, and the family.

### Study limitations

In order to assess the generalizability of our results for the overall Greek population, we compare the prevalence rates of different risk factors found in our sample with those of the population where nationwide recent data are available: We found that *adolescent pregnancy* in our sample (6.5%) is representative for the general population although the most recent prevalence rates available are reported for the year 2003 (5.6%) [94]. Smoking prevalence in our sample (15.1%) is representative of the pregnant population 17% [95]. Prevalence of *alcohol* consumption in the general population for the year 2011 was 8.2% [57] while 8.6% of our participants reported consuming alcohol during their gestation. *Immigrant population* is also representative in our sample; a 16% for both mothers and fathers, while in the general population the latest data in 2006 report a 10% of immigrants in Greece [96]. In our study 9.7% of the participants reported a history of psychiatric diagnosis. A much larger percentage (29%) reports that they sought psychological help at some point in their life. Similarly, in the study of Skapinakis, [97] 23.6% had visited a mental health professional in the past twelve months.

The prevalence of *physical violence and sexual abuse in childhood* as reported in studies for Greece have been somewhat confusing, while the latest available data are from 1997 report that around 11% of injured children brought for hospital attention are suspected to be a result of parents’ physical abuse while 5% is certain [98]. In our study 16% reported having experienced physical maltreatment during childhood. History of past *IPV* prevalence rates, were found to be slightly lower in our sample (2.2%) than in the general population (3.5%) [99] even though a much higher percentage (12.9%) reported having increased conflict and vociferous conflict (10.8%) with their partner in the past eight weeks.

To the best of our knowledge for all other risk factors assessed by the KINDEX no nationwide, representative data for (pregnant) women in Greece are available. But in general the comparison of our data with the prevalence rates of representative studies of the Greek population lead us to conclude that the study’s external validity is acceptable and the results can be cautiously generalized for the overall Greek population. We also attempted to interpret the findings of this study keeping in mind the specific cultural context and characteristics of Cretan society.

Among the limitations of this study is the use of scales that have not been validated in the general Greek population even though we followed strict back-translation procedure guidelines.

In the present study all results are only based on interviews that took place during pregnancy. For further studies we would recommend also follow-up measures in order to assess the adequate referral strategies and support network for the pregnant and maybe also to include observational means to assess the mother-child-interaction once the baby is born. As mentioned above adequate and low-threshold but evidence based intervention and prevention programs for pregnant women and mothers with babies and toddlers have to be developed and the preparedness of mental health and social services to handle cases of high-risk pregnant women should be enhanced. The importance of adequate interventions in this early stage should be raised not only in health professionals but also in politics, so that women in need do not pass-by unattended.

Thus, the external validity of the study and further generalizability of the results could be enhanced through further studies in diverse socio-economical contexts around Greece.

### Endnotes

^a^On Monday the first pregnant woman, on Tuesday the second, etc.

^b^http://www.forschung.uni-konstanz.de/en/research-support/guidelines-for-proposal-writing/general-guidelines/ethics-committee/.
